# Valenced action/inhibition learning in humans is modulated by a genetic variant linked to dopamine D2 receptor expression

**DOI:** 10.3389/fnsys.2014.00140

**Published:** 2014-08-06

**Authors:** Anni Richter, Marc Guitart-Masip, Adriana Barman, Catherine Libeau, Gusalija Behnisch, Sophia Czerney, Denny Schanze, Anne Assmann, Marieke Klein, Emrah Düzel, Martin Zenker, Constanze I. Seidenbecher, Björn H. Schott

**Affiliations:** ^1^Department of Neurochemistry and Molecular Biology, Department of Behavioral Neurology, Leibniz Institute for NeurobiologyMagdeburg, Germany; ^2^Wellcome Trust Centre for Neuroimaging, Institute of Neurology, University College LondonLondon, UK; ^3^Ageing Research Centre, Karolinska InstituteStockholm, Sweden; ^4^Institute of Human Genetics, Otto von Guericke University of MagdeburgMagdeburg, Germany; ^5^Institute of Cognitive Neurology and Dementia Research, Otto von Guericke University MagdeburgMagdeburg, Germany; ^6^Institute of Cognitive Neuroscience, University College LondonLondon, UK; ^7^German Center for Neurodegenerative DiseasesMagdeburg, Germany; ^8^Center for Behavioral Brain Sciences, Otto von Guericke University of MagdeburgMagdeburg, Germany; ^9^Department of Psychiatry, Charité University HospitalBerlin, Germany; ^10^Department of Neurology, University of MagdeburgMagdeburg, Germany

**Keywords:** dopamine D2 receptor, TaqIA, reward learning, motivated learning, action bias

## Abstract

Motivational salience plays an important role in shaping human behavior, but recent studies demonstrate that human performance is not uniformly improved by motivation. Instead, action has been shown to dominate valence in motivated tasks, and it is particularly difficult for humans to learn the inhibition of an action to obtain a reward, but the neural mechanism behind this behavioral specificity is yet unclear. In all mammals, including humans, the monoamine neurotransmitter dopamine is particularly important in the neural manifestation of appetitively motivated behavior, and the human dopamine system is subject to considerable genetic variability. The well-studied TaqIA restriction fragment length polymorphism (rs1800497) has previously been shown to affect striatal dopamine metabolism. In this study we investigated a potential effect of this genetic variation on motivated action/inhibition learning. Two independent cohorts consisting of 87 and 95 healthy participants, respectively, were tested using the previously described valenced go/no-go learning paradigm in which participants learned the reward-associated no-go condition significantly worse than all other conditions. This effect was modulated by the TaqIA polymorphism, with carriers of the A1 allele showing a diminished learning-related performance enhancement in the rewarded no-go condition compared to the A2 homozygotes. This result highlights a modulatory role for genetic variability of the dopaminergic system in individual learning differences of action-valence interaction.

## Introduction

Efficient decision making requires an individual to select responses that maximize reward and minimize punishment or loss. Such motivated behavior involves two fundamental axes of control, namely valence—spanning reward and punishment, and action—spanning invigoration and inhibition. Previous studies have shown that these two axes are not independent (Guitart-Masip et al., 2012b, 2013; Cavanagh et al., 2013; Chowdhury et al., 2013; for review see Guitart-Masip et al., 2014) and that decision making is not only influenced by an instrumental controller that learns to optimize choices on the basis of their contingent consequences, but also on a Pavlovian controller that generates stereotyped, “hard-wired” behavioral responses to the occurrence of motivationally salient outcomes or learned predictions of such outcomes (Dickinson and Balleine, 2002; Guitart-Masip et al., 2013). The presence of such “hard-wired” response patterns may be an evolutionarily beneficial adaptation to an environment world in which obtaining a reward typically requires some sort of overt behavioral response (*go to win*) whereas avoiding a punishment rather requires an avoidance of those actions that may lead to it (*no-go to avoid losing*). On the other hand, such a response bias may also be a source of suboptimal behavior when Pavlovian and instrumental controllers are in opposition (Breland and Breland, 1961; Dayan et al., 2006; Boureau and Dayan, 2011).

In order to manipulate action and valence orthogonally, Guitart-Masip et al. (2012b) designed a go/no-go learning task that involves besides the commonly investigated conditions *go to win* and *no-go to avoid losing* also the *vice versa* conditions where the participant needs to perform an action to avoid a punishment (*go to avoid losing*) or to inhibit an action to obtain a reward (*no-go to win*). Studies employing this task have repeatedly shown that while active choices in rewarded conditions and passive choices in punished conditions can be learned easily, it is significantly harder to learn an approach behavior to avoid a punishment and yet even more difficult to inhibit an action to obtain a reward. This asymmetry indicates that signals that predict reward are prepotently associated with behavioral activation, whereas signals that predict punishment are intrinsically coupled to behavioral inhibition.

In search for neural mechanisms underlying this behavioral asymmetry in the coupling between action and valence, monoaminergic, particularly dopaminergic, neuromodulation is a prime candidate (Gray and McNaughton, 2000; Boureau and Dayan, 2011; Cools et al., 2011). Dopamine (DA) is believed to enable or enhance the generation of active motivated behavior (Berridge and Robinson, 1998; Niv et al., 2007; Salamone et al., 2007; Beierholm et al., 2013) and to support instrumental learning (Frank et al., 2004; Daw and Doya, 2006; Wickens et al., 2007). It has been observed that DA depletion leads to decreased motor activity and decreased motivated behavior (Ungerstedt, 1971; Palmiter, 2008), along with decreased vigor or motivation to work for rewards in demanding reinforcement schedules (Salamone et al., 2005; Niv et al., 2007). Conversely, boosting DA levels with levodopa invigorates motor responses in healthy humans (Guitart-Masip et al., 2012a) and DA promotes “go” and impairs “no-go” learning, for example in patients with Parkinson's disease (Frank et al., 2004). However, contrary to the expectations suggested by this evidence, administration of levodopa reduced the learning disadvantage of the *no-go to win* condition when compared to the *no-go to avoid losing* (Guitart-Masip et al., 2013). These effects suggested that DA is involved in decreasing the coupling between action and valence, supposedly via DA's actions on neural functions implemented in prefrontal cortex (Hitchcott et al., 2007). It is therefore unclear how striatal DA modulates the coupling between action and valence uncovered in this task.

The aim of the present study was to test whether naturally occurring differences in healthy humans in this valenced action/inhibition learning might arise from dopaminergic mechanisms and how striatal DA effects the action/valence interaction. To address this issue, we used the valenced go/no-go learning paradigm in a cohort of young, healthy subjects, and tested them for the TaqIA restriction length polymorphism (rs1800497), a common genetic variation of the dopamine D2 receptor (DRD2) gene known to affect D2 receptor expression and striatal DA metabolism. Although the underlying molecular mechanisms are yet not fully understood, the TaqIA polymorphism has been repeatedly associated with reduced striatal DRD2 density in A1 carriers as evident from three *post mortem* studies (Noble et al., 1991; Thompson et al., 1997; Ritchie and Noble, 2003) and two out of three conducted *in vivo* binding studies (Laruelle et al., 1998; Pohjalainen et al., 1998; Jonsson et al., 1999). Laakso et al. (2005) suggested that the lower D2 receptor expression leads to decreased autoreceptor function, thereby increasing the DA and/or trace amine synthesis rate in the brains of A1 allele carriers. Moreover, Kirsch et al. (2006) observed an increase of striatal BOLD signal in response to the dopamine D2 receptor agonist bromocriptine in subjects carrying the A1 allele, but not in subjects without the A1 allele, and Stelzel et al. (2010) reported a generally increased striatal BOLD signal in A1 carriers. As striatal BOLD signal has been shown to correlate with DA release (Schott et al., 2008), the increased striatal activation in A1 carriers might be related to higher presynaptic dopaminergic activity (Richter et al., 2013). Because striatal DA is associated with linking action with reward (Berridge and Robinson, 1998; Frank et al., 2004; Daw and Doya, 2006; Niv et al., 2007; Salamone et al., 2007; Wickens et al., 2007; Beierholm et al., 2013), we hypothesized that A1 carriers might show increased coupling between action and valence.

## Materials and methods

### Participants

Participants were recruited from a cohort of 719 young healthy volunteers of Caucasian ethnicity of a large-scale behavioral genetic study conducted at the Leibniz Institute for Neurobiology, Magdeburg. Given our hypothesis regarding differential performance in the valenced go/no-go task as a function of striatal D2 receptor availability, we selected participants a priori as a function of DRD2 TaqIA genotype. To control for confounding effects of genetic influences on prefrontal DA availability, we also ensured a balanced distribution of the COMT Val108/158 Met polymorphism that is known to affect prefrontal DA levels and D1 receptor binding (Gogos et al., 1998; Matsumoto et al., 2003; Meyer-Lindenberg et al., 2005; Slifstein et al., 2008). All participants were right-handed according to self-report, not genetically related, and had obtained at least a university entrance diploma (Abitur) as educational certificate. Importantly, all participants had undergone routine clinical interview to exclude present or past neurological or psychiatric illness, alcohol, or drug abuse, use of centrally acting medication, the presence of psychosis or bipolar disorder in a first-degree relative, and additionally, given the design of the experiment, regular gambling. Two independent cohorts of healthy participants were tested (cohort 1: 43 females and 44 males; age: range 19–36 years, mean 24.6 years, *SD* = 3.1 years; cohort 2: 48 females and 47 males; age: range 20–33 years, mean 24.6 years, *SD* = 2.8 years). Because of a previously reported potential association of the A1 allele with nicotine consumption (Verde et al., 2011; for reviews see Comings and Blum, 2000; Lerman et al., 2007), smoking status was assessed from the participants. All participants gave written informed consent in accordance with the Declaration of Helsinki and received financial compensation for participation. The work was approved by the Ethics Committee of the University of Magdeburg, Faculty of Medicine.

### Genotyping

The DRD2/ANKK1 TaqIA restriction length polymorphism (NCBI accession number: rs1800497) was genotyped using a protocol previously described in Richter et al. (2013). Genomic DNA was extracted from blood leukocytes using the GeneMole^®^ automated system (Mole Genetics AS, Lysaker, Norway) according to the manufacturer's protocol. Genotyping was performed using PCR followed by allele-specific restriction analysis using previously described primers (Grandy et al., 1989). Genotyping was also performed for several additional polymorphisms, including COMT Val108/158 Met (see Table 1), to control for confounding effects of other genetic variants and to reduce the risk of population stratification.

**Table 1:** **Genotyped polymorphisms**.

**Polymorphism/Gene**	**NCBI accession number**	**Genotyping protocol**
DRD2/ANKK1 TaqIA	rs1800497	Richter et al., 2013
		Primers for PCR:
		5′-CCGTCGACGGCTGGCCAAGTTGTCTA-3′
		5′-CCGTCGACCCTTCCTGAGTGTCATCA-3′
		Restriction enzyme: TaqI
COMT Val108/158 Met	rs4680	Schott et al., 2006; Wimber et al., 2011
		Primers for PCR:
		5′-ATGGCCCGCCTGCTGTCACCAG-3′
		5′-TCTGACAACGGGTCAGGCACGCACAC-3′
		Restriction enzyme: Hin1ll (NlaIII)
DAT1 VNTR	rs28363170	Schott et al., 2006
		Primers for PCR:
		5′-TGTGGTGTAGGAAACGGCCTGAG-3′
		5′-CTTCCTGGAGGTCACGGCTCAAAGG-3′
		PCR products were not digested
DRD2 C957T	rs6277	*Kompetitive allele-specific PCR (KASP)*
		Assay on Demand (LGC Genomics, Berlin, Germany)
DARPP-32	rs907094	Primers for PCR:
		5′-GCACCCCATGGAGCGAGAAGACAG-3′
		5′-CGCATTGCTGAGTCTCACCTGCAGTC-3′
		Restriction enzyme: Tru1l

### Paradigm

**Figure 1:**
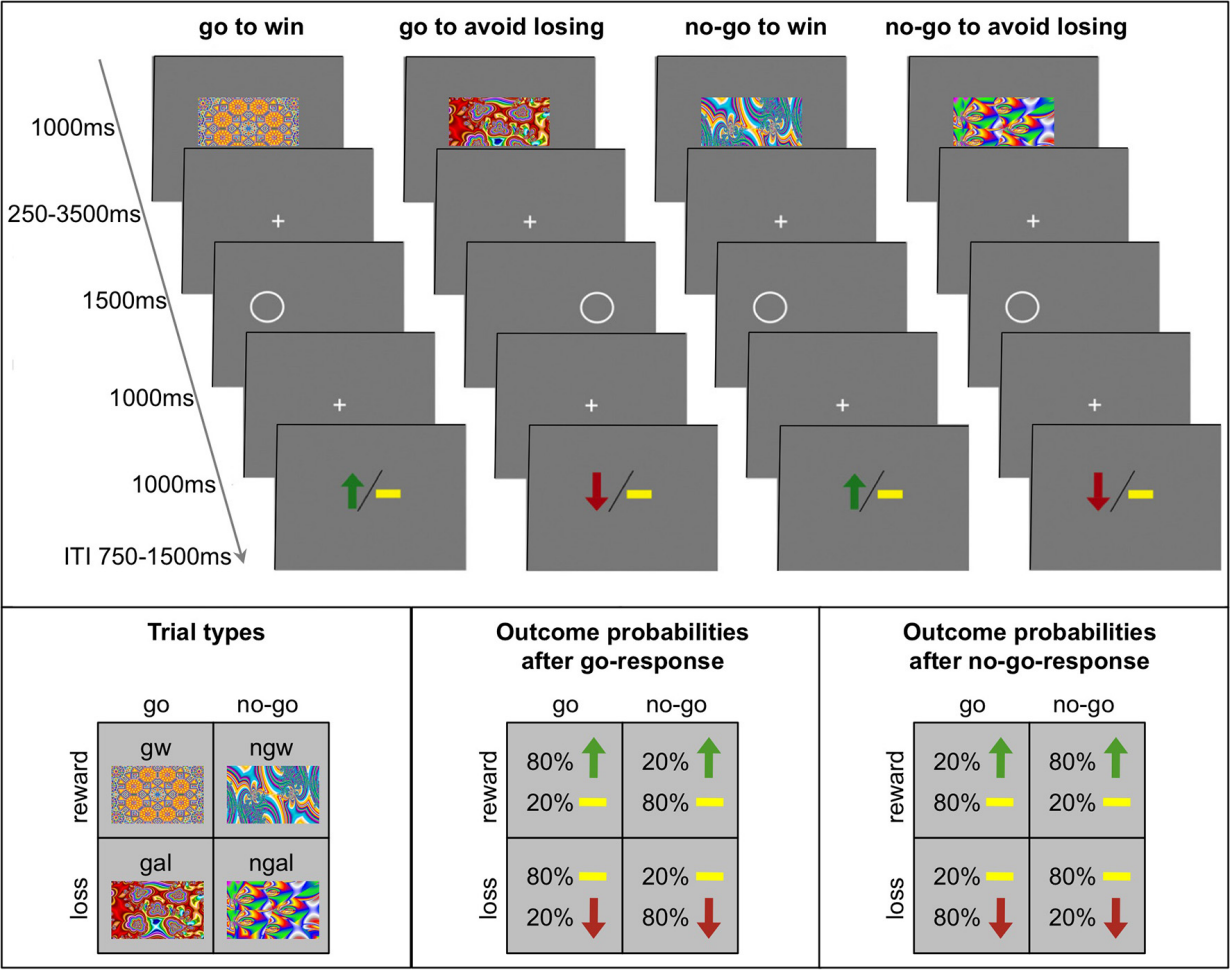
**Experimental paradigm of the probabilistic monetary go/no-go task**. Fractal images indicate the combination between action (go or no-go) and valence (reward or loss). On go trials, subjects press a button for the side of a circle. On no-go trials they withhold a response. Arrows indicate rewards (green) or losses (red). Horizontal bars (yellow) symbolize the absence of a win or a loss. The schematics at the bottom represent for each trial type the nomenclature (left), the possible outcomes and their probabilities after response to the target (“go”; middle), and the possible outcomes and their probability after withholding a response to the target (“no-go”; right). gw, go to win; gal, go to avoid losing; ngw, no-go to win; ngal, no-go to avoid losing; ITI, intertrial interval.

We used a previously employed go/no-go learning task with orthogonalized action requirements and outcome valence (Guitart-Masip et al., 2012b, 2013; Chowdhury et al., 2013). The trial timing is displayed in Figure 1. Each trial consisted of presentation of a fractal cue, a target detection task, and a probabilistic outcome. First, one out of four abstract fractal cues was displayed for 1000 ms. Participants were informed that a fractal indicated whether they would subsequently be required to perform a target detection task by pressing a button (go) or not (no-go) and that the cue also indicated the possible valence of the outcome of the subjects' behavior (reward/no reward or punishment/no punishment). However, subjects were not instructed about the contingencies for each fractal image and had to learn them by trial and error. The meaning of the fractal images was randomized across participants. Following a variable interval (250–3500 ms) after offset of the fractal image, the target detection task started: participants had the opportunity to press a button within a time limit of 2000 ms to indicate the side of a circle for go trials, or not to press for no-go trials. After the offset of the circle after 1500 and 1000 ms of fixation, subjects were presented with the outcome. The outcome remained on screen for 2000 ms and after a variable intertrial interval (ITI; 750–1500 ms) a new trial started. Participants were informed that the outcome was probabilistic: in *win* trials 80% of correct choices and 20% of incorrect choices were rewarded with 0.50 € (the remaining 20% of correct and 80% of incorrect choices leading to no outcome), while in *avoid losing* trials 80% of correct choices and 20% of incorrect choices avoided a loss of 0.50 € (the remaining 20% of correct and 80% of incorrect choices leading to a punishment). Thus, there were four trial types depending on the nature of the fractal cue presented at the beginning of the trial: press the correct button in the target detection task to gain a reward (*go to win*); press the correct button in the target detection task to avoid punishment (*go to avoid losing*); do not press a button in the target detection task to gain a reward (*no-go to win*); do not press a button in the target detection task to avoid punishment (*no-go to avoid losing*). The task included 240 trials, 60 trials per condition and was divided into four sessions 9 min each (15 trials per condition in randomized order). Subjects were told that they would be paid their earnings of the task up to a total of 25 € and a minimum of 7 €. Before starting with the learning task, subjects performed 10 trials of the target detection task in order to get familiarized with the speed requirements.

### Statistical analysis

The percentage of correct choices in the target detection task (correct button press for go conditions and correct omission of responses in no-go trials) was collapsed across time bins of 30 trials per condition and analyzed with a mixed ANOVA with time (1st/2nd half), action (go/no-go), and valence (win/lose) as within-subject factors and TaqIA genotype (A1+/A1−) as between-subject factor. Additionally reaction times of correct go responses (RTs) were analyzed using a mixed ANOVA with valence (win/lose) and TaqIA genotype (A1+/A1−) as factors. When appropriate, paired *t*-test, independent sample *t*-test or Mann-Whitney *U*-test were used as *post-hoc* tests.

The analysis of the behavioral data was done in two stages. In cohort 1 we included the TaqIA and the COMT Val108/158 Met polymorphism as between-subject factors. In the second we specifically aimed to replicate the significant effect of TaqIA. The following statistics include TaqIA as the only between-subject factor.

## Results

### Genotyping

Genotyping was performed in the entire cohort of 719 subjects, and two sub-cohorts were recruited based on the DRD2/ANKK1 TaqIA genotype. The data of 87 participants in cohort 1 and 95 participants in cohort 2 were analyzed. In cohort 1, we identified 4 A1 homozygotes, 33 heterozygotes and 50 A2 homozygotes. In cohort 2, genotyping revealed 4 A1 homozygotes, 30 heterozygotes and 61 A2 homozygotes. The distributions in both groups were at Hardy-Weinberg equilibrium (cohort 1: χ^2^ = 0.24, *p* = 0.621; cohort 2: χ^2^ = 0.02, *p* = 0.898). A1 carriers (A1+: A1/A1 and A1/A2) were grouped together for all subsequent analyses as in previous behavioral and imaging studies of the TaqIA polymorphism (Stelzel et al., 2010; Richter et al., 2013). The groups A1+ and A1− (A2/A2) did not differ in gender, in age or in the number of smokers and nonsmokers (Table 2).

**Table 2:** **Demographic data**.

	**A1+**	**A1−**	
**COHORT 1**			
Women/Men (*n* = 87)	17/20	26/24	χ^2^ = 0.31, *p* = 0.577
Mean age (*n* = 87)	24.9 ± 3.6	24.3 ± 2.6	*t*_(85)_ = 0.83, *p* = 0.410
Smokers/Nonsmokers (*n* = 87)	15/22	14/36	χ^2^ = 1.51, *p* = 0.220
COMT mm/vm/vv (*n* = 87)	13/14/10	18/15/17	χ^2^ = 0.73, *p* = 0.694
DAT1-VNTR 9+/9− (*n* = 85)	11/25	15/34	χ^2^ < 0.01, *p* = 0.996
C957T CC/CT/TT (*n* = 87)	11/19/7	8/24/18	χ^2^ = 4.04, *p* = 0.132
DARPP-32 CC/CT/TT (*n* = 87)	20/15/2	29/19/2	χ^2^ = 0.19, *p* = 0.912
**COHORT 2**			
Women/Men (*n* = 95)	13/21	35/26	χ^2^ = 3.20, *p* = 0.074
Mean age (*n* = 95)	25.2 ± 3.3	24.2 ± 2.4	*t*_(93)_ = 1.58, *p* = 0.121
Smokers/Nonsmokers (*n* = 95)	5/29	14/47	χ^2^ = 0.93, *p* = 0.335
COMT mm/vm/vv (*n* = 95)	11/14/9	19/27/15	χ^2^ = 0.09, *p* = 0.957
DAT1-VNTR 9+/9− (*n* = 93)	17/17	32/27	χ^2^ = 0.16, *p* = 0.693
C957T CC/CT/TT (*n* = 95)	15/17/2	3/37/21	χ^2^ = 25.49, *p* < 0.001
DARPP-32 CC/CT/TT (*n* = 95)	15/16/3	41/20/0	χ^2^ = 0.8.53, *p* = 0.014

Gender distribution, age (means ± standard deviations), number of smokers and nonsmokers. Allelic distributions for following polymorphisms: COMT Val108/158 Met (mm, met homozygotes; vm, val/met heterozygotes; mm, met homozygotes), DAT1-VNTR (9+, carriers of the 9-repeat allele 9/9 and 9/10; 9−, 10-repeat homozygous subjects 10/10), C957T (CC/CT/TT carriers) and DARPP-32 (CC/CT/TT carriers). A1+; carriers of the A1 allele. A1−; A2 homozygotes.

To control for effects of prefrontal DA availability, participants were also selected regarding the COMT Val108/158 Met (NCBI accession number: rs4680) polymorphism. Genotyping revealed 31 Met/Met, 29 Val/Met, and 27 Val/Val carriers in cohort 1 and 30 Met/Met, 41 Val/Met, and 24 Val/Val carriers in cohort 2. Allelic distribution for the COMT Val108/158 Met polymorphism did not differ significantly for either TaqIA A1 carriers or A2 homozygotes (Table 2). The experimenters who performed the behavioral task were blinded regarding DRD2/ANKK1 and COMT genotypes.

To further control for effects of population stratification and potential effects of putatively functional genetic variations in the dopamine system, genotyping was also performed for the DAT1-VNTR (NCBI accession number: rs28363170), the C957T polymorphism within the DRD2 gene (NCBI accession number: rs6277) and the DARPP-32 polymorphism (NCBI accession number: rs907094) (see Table 1). Allelic distributions for the DAT1-VNTR polymorphism did not differ significantly for either TaqIA A1 carriers or A2 homozygotes (Table 2). However, because of differences for the C957T and the DARPP-32 polymorphism, we additionally calculated an ANCOVA including these two polymorphisms as covariates (see below).

### Behavioral results

We initially performed an omnibus mixed-design ANOVA to test for effects of both DRD2/ANKK1 and COMT genotypes. There was a significant four-fold interaction of DRD2/ANKK1 TaqIA with action, time and valence [*F*_(1,81)_ = 5.11, *p* = 0.027], but no effect of COMT Val108/158 Met polymorphism (all *p* > 0.120). All further analyses were therefore focused on the DRD2/ANKK1 TaqIA polymorphism. We computed as ANOVA for repeated measures on the percentage of correct (optimal) choices with action (go/no-go), valence (win/lose) and time (1st/2nd half) as within-subject factors and genotype (A1+/A1−) as between-subject factor. See Table 3 for statistics.

**Table 3:** **Statistics on percentage of correct responses**.

**Effects**	**Cohort 1**	**Cohort 2**
Action	*F*_(1, 85)_ = 62.56, *p* < 0.001, η^2^ = 0.42	*F*_(1, 93)_ = 50.87, *p* < 0.001, η^2^ = 0.35
Go > no-go	go: = 87 ± 12% no-go: = 73 ± 21% *t*_(86)_ = 7.97, *p* < 0.001	go: = 91 ± 9% no-go: = 79 ± 18% *t*_(94)_ = 7.68, *p* < 0.001
Time	*F*_(1, 85)_ = 135.92, *p* < 0.001, η^2^ = 0.62	*F*_(1, 93)_ = 189.21, *p* = < 0.001, η^2^ = 0.67
2nd half > 1st half	1st half: = 74 ± 15% 2nd half: = 86 ± 16% *t*_(86)_ = 11.89, *p* < 0.001	1st half: = 78 ± 13% 2nd half: = 92 ± 13% *t*_(94)_ = 14.68, *p* < 0.001
Action × valence	*F*_(1, 85)_ = 44.41, *p* < 0.001, η^2^ = 0.34	*F*_(1, 93)_ = 37.72, *p* < 0.001, η^2^ = 0.29
Go to win > go to avoid losing	gw: = 91 ± 14% gal: = 82 ± 14% *t*_(86)_ = 6.28, *p* < 0.001	gw: = 95 ± 12% gal: = 87 ± 10% *t*_(94)_ = 5.74, *p* < 0.001
No-go to avoid losing > no-go to win	ngw: = 66 ± 32% ngal: = 81 ± 16% *t*_(86)_ = 4.99, *p* < 0.001	ngw: = 73 ± 30% ngal: = 86 ± 11% *t*_(94)_ = 4.63, *p* < 0.001
Action × time	*F*_(1, 85)_ = 19.09, *p* < 0.001, η^2^ = 0.18	*F*_(1, 93)_ = 59.77, *p* < 0.001, η^2^ = 0.39
1st half(go—no-go) > 2nd half(go—no-go)	1st half: = 17 ± 17% 2nd half: = 9 ± 18% *t*_(86)_ = 4.62, *p* < 0.001	1st half: = 18 ± 17% 2nd half: = 6 ± 16% *t*_(94)_ = 8.46, *p* < 0.001
Action × valence × time × genotype	*F*_(1, 85)_ = 5.24, *p* = 0.025, η^2^ = 0.06	*F*_(1, 93)_ = 4.59, *p* = 0.035, η^2^ = 0.05
A1−(ngw(2nd—1st half)) > A1+(ngw(2nd—1st half))	A1+: = 8 ± 21% A1−: = 22 ± 26% *t*_(85)_ = 2.78, *p* = 0.007	A1+: = 15 ± 22% A1−: = 25 ± 24% *t*_(93)_ = 2.16, *p* = 0.033

Means ± standard deviations are shown. Only effects that were significant in both cohorts are reported. ANOVA was computed with percent correct responses as dependent variable and action, valence, time and genotype as independent variables. Paired t-tests and t-tests for independent samples were performed as post-hoc tests. gw, go to win; gal, go to avoid losing; ngw, no-go to win; ngal, no-go to avoid losing. A1+; carriers of the A1 allele. A1−; A2 homozygotes.

Our study reproduced a main effect of action [cohort 1: *F*_(1, 85)_ = 62.56, *p* < 0.001; cohort 2: *F*_(1, 93)_ = 50.87, *p* < 0.001] and an action by valence interaction [cohort 1: *F*_(1, 85)_ = 44.41, *p* < 0.001; cohort 2: *F*_(1, 93)_ = 37.72, *p* < 0.001], as demonstrated in previous studies (Guitart-Masip et al., 2012b, 2013; Cavanagh et al., 2013; Chowdhury et al., 2013). Subjects showed better performance in conditions requiring a go choice than in trials requiring a no-go choice [cohort 1: *t*_(86)_ = 7.97, *p* < 0.001; cohort 2: *t*_(94)_ = 7.68, *p* < 0.001], and while they were better at learning from reward as compared to punishment in the go condition [cohort 1: *t*_(86)_ = 6.28, *p* < 0.001; cohort 2: *t*_(94)_ = 5.74, *p* < 0.001], this relation reversed in the no-go condition [cohort 1: *t*_(86)_ = 4.99, *p* < 0.001; cohort 2: *t*_(94)_ = 4.63, *p* < 0.001]. As Guitart-Masip et al. (2012b, 2013) we also observed a main effect of time [cohort 1: *F*_(1, 85)_ = 135.92, *p* < 0.001; cohort 2: *F*_(1, 93)_ = 189.21, *p* =< 0.001] and additionally an action by time interaction [cohort 1: *F*_(1, 85)_ = 19.09, *p* < 0.001; cohort 2: *F*_(1, 93)_ = 59.77, *p* < 0.001], indicating a preponderant initial bias toward go responses [cohort 1: *t*_(86)_ = 4.62, *p* < 0.001; cohort 2: *t*_(94)_ = 8.46, *p* < 0.001].

**Figure 2:**
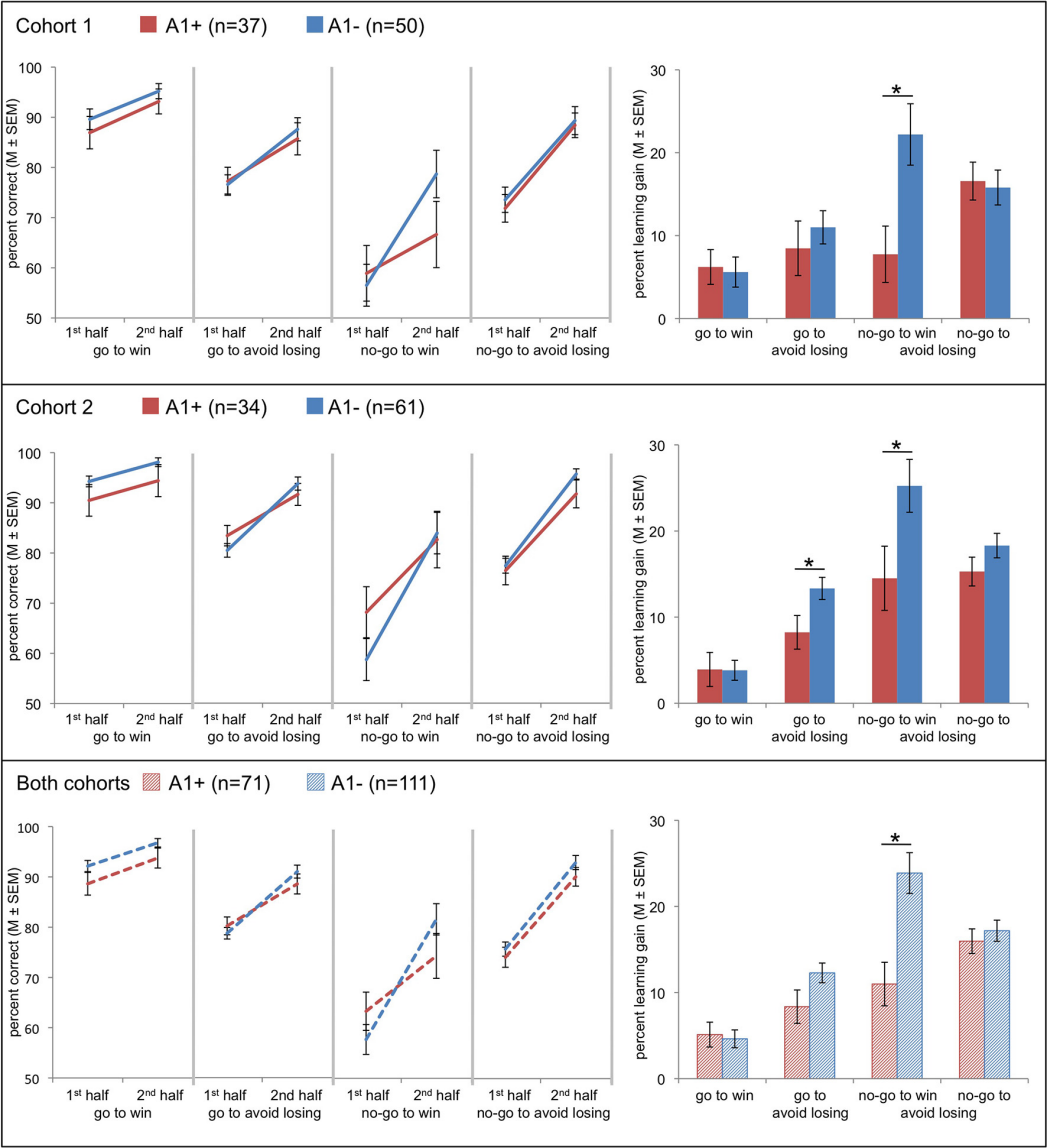
**Effects of Taq1A genotype on choice performance in two independent cohorts and in the entire sample (data of both cohorts combined)**. Line charts at the left show mean values of correct responses (±s.e.m.) in A1 carriers (red) and A2 homozygotes (blue) in the first and the second half of trials for all four conditions. Bar plots at the right show the differences between mean (±s.e.m.) values of correct responses of second half of trials minus first half of trials in A1 carriers (red) and A2 homozygotes (blue) for each condition. This score represents the four-fold interaction of action by valence by time by genotype. Compared to the A2 homozygotes carriers of the A1 allele showed a diminished learning to withhold an action to receive a reward. *Post-hoc* comparisons via *t*-test: ^*^p < 0.05.

Most interestingly for the current study, we observed a four-fold interaction of action by valence by time by genotype [cohort 1: *F*_(1, 85)_ = 5.24, *p* = 0.025; cohort 2: *F*_(1, 93)_ = 4.59, *p* = 0.035]. This effect was observed in the absence of an action by valence by genotype effect (cohort 1: *p* = 0.811; cohort 2: *p* = 0.087). While the genotype groups did not differ significantly in their mean performance in the first and second time bin in any condition (cohort 1: *p* > 0.143; cohort 2: *p* > 0.167), they showed a different degree of improvement from the first to the second time interval (learning gain: mean performance 2nd half—mean performance 1st half; see Figure 2). Performance of the A2 homozygotes in the *no-go to win* condition showed increased improvement from the first to the second half of the experiment compared to the A1 carriers [cohort 1: *t*_(85)_ = 2.78, *p* = 0.007]. In the second cohort this result was replicated [cohort 2: *t*_(93)_ = 2.16, *p* = 0.033], and A1 carriers showed lower performance in the *go to avoid losing* condition [cohort 2: *t*_(93)_ = 2.26, *p* = 0.026]. Because performance in the *no-go to win* condition during early trials differed between the two cohorts, we tested whether the observed interaction, which would likely reflect a difference in learning rate, remained significant when combining both datasets. A Three-Way ANCOVA across both cohorts (including cohort as a covariate of no interest; see Figure 2) revealed the same three-way interaction revealed by the analyses in the separate cohorts [*F*_(1, 179)_ = 9.87, *p* = 0.002]. Only in one cohort there was a statistically significant three-way interaction [action by valence by time; cohort 1: *F*_(1, 85)_ = 0.42, *p* = 0.517; cohort 2: *F*_(1, 93)_ = 10.98, *p* = 0.001] and a time by genotype interaction [cohort 1: *F*_(1, 85)_ = 3.77, *p* = 0.055; cohort 2: *F*_(1, 93)_ = 6.31, *p* = 0.014].

Statistics regarding reaction times (RTs) of the go responses are summarized in Table 4. We computed an ANOVA with valence (win/lose) as within-subject factor and genotype as between-subject factor. Irrespective of genotype, RTs in the *go to win* condition were shorter than in the *go to avoid losing* condition [cohort 1: *F*_(1, 85)_ = 14.06, *p* < 0.001; cohort 2: *F*_(1, 93)_ = 11.21, *p* = 0.001]. Regarding DRD2/ANKK1 TaqIA genotype, there was only a trendwise interaction with valence [*F*_(1, 93)_ = 3.38, *p* = 0.069] and a trend for a main effect [*F*_(1, 93)_ = 3.67, *p* = 0.058] in cohort 2, with the A1 carriers being slower in avoiding punishment as compared to the A2 homozygotes [*t*_(93)_ = 2.04, *p* = 0.046]. Although this nominal effect together with the worse accuracy of the A1 carriers in the *go to avoid losing* condition (Figure 2) hints at a worse performance of the A1 carriers in this condition, the interpretation of this result warrants caution as the effects were only apparent in cohort 2 and, moreover, participants were explicitly instructed to respond accurately, while speed was not emphasized.

**Table 4:** **Statistics on reaction times of correct go responses**.

	**A1+**	**A1−**	
**COHORT 1**			
Go to win	527 ± 128 ms	535 ± 88 ms	*t*_(85)_ = −0.36, *p* = 0.719
Go to avoid losing	547 ± 129 ms	564 ± 117 ms	*t*_(85)_ = −0.65, *p* = 0.521
**COHORT 2**			
Go to win	561 ± 100 ms	534 ± 76 ms	*t*_(93)_ = 1.48, *p* = 0.144
Go to avoid losing	583 ± 107 ms	540 ± 76 ms	*t*_(93)_ = 2.04, *p* = 0.046

Means ± standard deviations are shown. A1+; carriers of the A1 allele. A1−; A2 homozygotes.

To rule out that the genotype effects are not simply explained by differences in target detection performance the percentage of trials in which subjects responded incorrectly in the target detection task (i.e., left when the target was on the right side of the display or vice versa) was measured and did not differ significantly between genotype groups (Mann-Whitney *U*-test: cohort 1: A1+: *M* ± *SD* = 1 ± 3%, A1−: *M* ± *SD* = 1 ± 2%, *z* = −0.334, *p* = 0.738; cohort 2: A1+: *M* ± *SD* = 1 ± 3%, A1−: *M* ± *SD* = 0 ± 1%, *z* = −0.428, *p* = 0.668).

Because the TaqIA polymorphism is located downstream of the DRD2 gene, the observed genotype effects might putatively result from linkage disequilibrium with other DRD2 polymorphisms, including the C957T. We indeed observed an imbalanced distribution of the C957T polymorphism (rs6277) among TaqIA A1 carriers vs. A2 homozygotes numerically in the first cohort (χ^2^ = 4.04, *p* = 0.132) and significantly in the second cohort (χ^2^ = 25.49, *p* < 0.001). Moreover, the DARPP-32 polymorphism (rs907094) was unequally distributed in the second cohort only (χ^2^ = 8.53, *p* = 0.014). In order to rule out confounding effects, we included the polymorphisms as covariates in an additional ANCOVA. The same was done for COMT Val108/158 Met (rs4680), because the cohorts were stratified with respect to that polymorphism. Importantly, the four-fold action by valence by time by genotype interaction for the TaqIA polymorphism remained significant [cohort 1: *F*_(1, 82)_ = 4.63, *p* = 0.034, cohort 2: *F*_(1, 90)_ = 5.07, *p* = 0.027], while there was no effect for C957T (cohort 1: *p* = 0.472, cohort 2: *p* = 0.810), DARPP-32 (cohort 1: *p* = 0.578, cohort 2: *p* = 0.148) or COMT Val108/158 Met polymorphism (cohort 1: *p* = 0.161, cohort 2: *p* = 0.856).

## Discussion

The goal of this study was to investigate how a genetic variant linked to striatal DA responsivity affects the action/valence interaction. To this end, two independent cohorts consisting of 87 and 95 healthy participants were genotyped for the well-characterized DRD2/ANKK1 TaqIA polymorphism (Grandy et al., 1989; Dubertret et al., 2004; Neville et al., 2004) and performed the previously described valenced go/no-go task (Guitart-Masip et al., 2012b, 2013, 2014; Cavanagh et al., 2013; Chowdhury et al., 2013). Our results show differential learning performance in the carriers of the less common A1 allele of the TaqIA polymorphism, which has previously been linked to lower striatal dopamine D2 receptor expression. Replicating previous results, participants were, irrespective of genotype, more successful in learning active choices in rewarded conditions and passive choices in punished conditions, with response inhibition to obtain a reward (*no-go to win*) being the condition most difficult to learn. The DRD2 TaqIA polymorphism exerted a modulatory influence on learning performance in the *no-go to win* condition with A1 carriers showing lower learning rates throughout the experiment.

It has to be emphasized that, despite the fact that in the present study learning curves of the two cohorts differed to some extent and initial performance of A1 carriers was not identical, we did yet observe a replicable attenuation of learning rates in A1 carriers that was specific to the *no-go to win* condition, and, importantly, the effect was even more pronounced when combining both datasets (using cohort as a covariate of no interest; see Figure 2).

**Figure 3:**
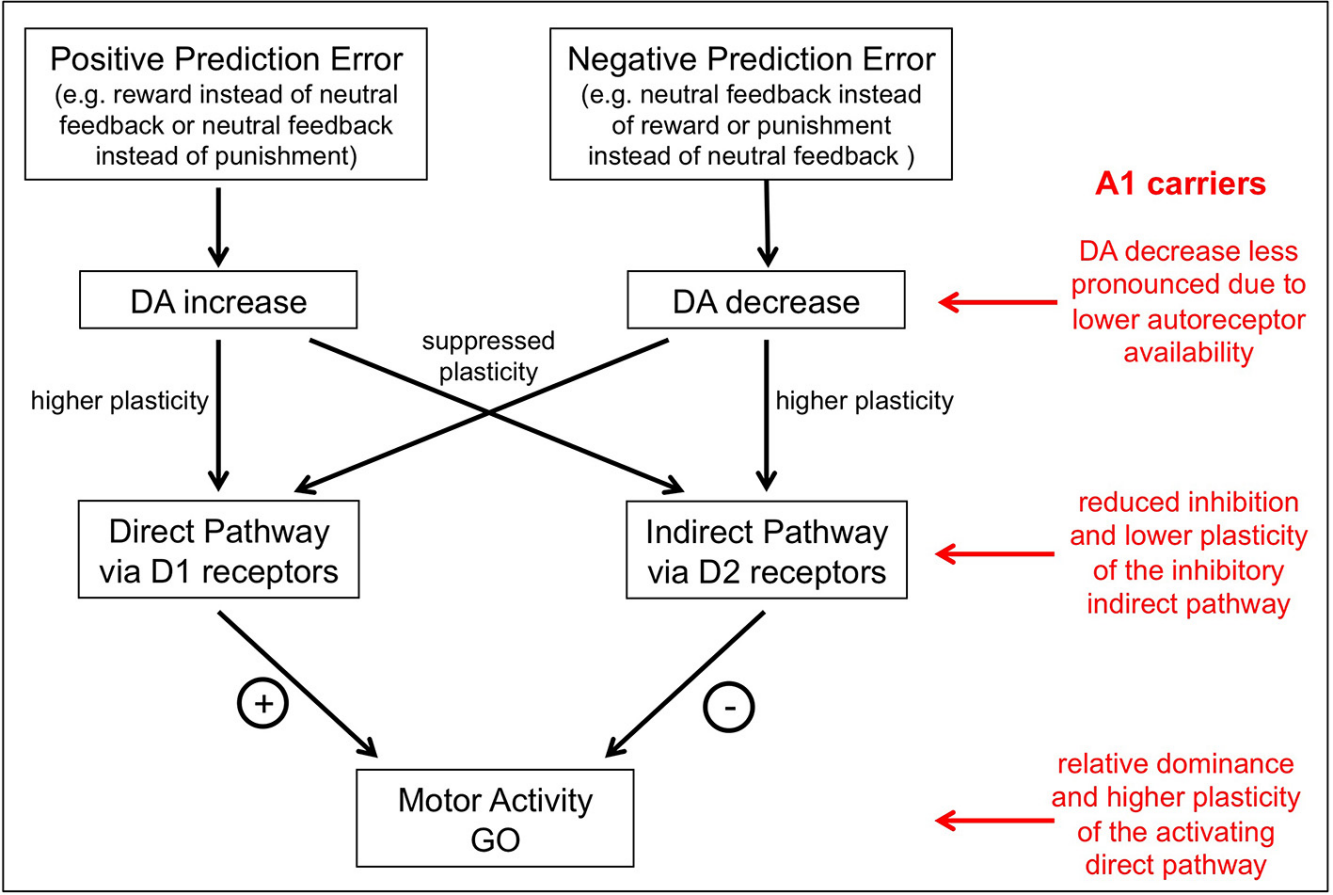
**A model of the putative influence of the TaqIA polymorphism on action-valence interaction**. DA neurons signal reward prediction errors in the form of phasic bursts for positive prediction errors and dips below baseline firing rate for negative prediction errors. Increases of DA in response to an unexpected reward reinforce the direct pathway via activation of D1 receptors and thereby facilitate the future generation of go choices under similar circumstances, while dips in DA levels in response to an unexpected punishment reinforce the indirect pathway via reduced activation of D2 receptors and thus facilitate the subsequent generation of no-go choices in comparable situations. A1 carriers have less D2 receptors and thus would be assumed to have less limitation of dopaminergic signaling after negative prediction errors in the indirect pathway and a shift to a more action-oriented behavioral pattern mediated by the direct pathway.

It is important to note that there are two potential mechanisms by which valence can disrupt the choice of appropriate actions in the current task. The first mechanism is implemented at the time of the choice and can be seen as “Pavlovian” mechanism by which the anticipation of reward or punishment promotes action or inhibition, respectively (Dayan et al., 2006; Huys et al., 2011; Guitart-Masip et al., 2012b). The second mechanism is implemented at the time of outcome and is related to the role of DA within the striatum. According to a prevalent view in reinforcement learning and decision making, DA neurons signal reward prediction errors (Montague et al., 1996; Schultz et al., 1997; Bayer and Glimcher, 2005), in the form of phasic bursts for positive prediction errors and dips below baseline firing rate for negative prediction errors (Bayer et al., 2007), resulting in corresponding peaks and dips of dopamine availability in target structures, most prominently the striatum (McClure et al., 2003; O'Doherty et al., 2003, 2004; Pessiglione et al., 2006). In the striatum, increases of DA in response to an unexpected reward reinforce the direct pathway via activation of D1 receptors and thereby facilitate the future generation of go choices under similar circumstances, while dips in DA levels in response to an unexpected punishment reinforce the indirect pathway via reduced activation of D2 receptors and thus facilitate the subsequent generation of no-go choices in comparable situations (Frank et al., 2004, 2007; Wickens et al., 2007; Hikida et al., 2010; see Figure 3).

The effects related to the TaqIA polymorphism observed in the present study apparently reflect changes in the learning process, thus likely pointing to the function of DA in the ability to flexibly learn go or no-go choices based on the outcomes produced by previous actions. Our results are in apparent contrast to the effects previously reported in the same task after administration of levodopa. In that study, boosting DA levels resulted in a decoupling between action and valence that did not reflect any changes in the rate of learning (Guitart-Masip et al., 2013). Instead, the effects observed in that study boosted the asymptote reached by the participants that received levodopa. Using computational modeling, that effect was best characterized as a decreased influence of a Pavlovian control mechanism over the instrumental control mechanisms attempting to learn the task (Guitart-Masip et al., 2013). Similarly, in older adults, structural MRI measures of substantia nigra/ventral tegmental area (SN/VTA) integrity have also been linked to improved learning and a lower action bias (Chowdhury et al., 2013). One proposed explanation for the reduced coupling between action and valence in conditions associated with increased DA availability has been a likely increase of dopaminergic activity in the prefrontal cortex where DA influences the balance between different control mechanisms (Hitchcott et al., 2007). The implication of a prefrontal mechanism decreasing the Pavlovian influences on behavior and supporting performance of the *no-go to win* condition in this task has been shown in fMRI (Guitart-Masip et al., 2012b) and EEG experiments (Cavanagh et al., 2013). It should be noted, though, that, in the present study, we did not observe any behavioral differences as a function of the COMT Val108/158 Met polymorphism, which has previously been linked to prefrontal dopamine availability (Meyer-Lindenberg et al., 2005).

Receptor binding studies *in vitro* and *in vivo* have shown that A1 carriers show lower striatal D2 receptor expression (Noble et al., 1991; Thompson et al., 1997; Pohjalainen et al., 1998; Jonsson et al., 1999; Ritchie and Noble, 2003). On the other hand, A1 carriers also exhibit increased striatal DA synthesis, possibly as a result of reduced autoinhibitory signaling from presynaptic D2-type autoreceptors (Laakso et al., 2005). Previous behavioral and neuroimaging studies have in fact yielded results that would be best explained by parallel reduction of striatal postsynaptic D2 receptors and increased presynaptic dopaminergic activity in A1 carriers, with the latter also resulting in increased DA availability both in the striatum and in extrastriatal regions (Kirsch et al., 2006; Stelzel et al., 2010; Richter et al., 2013). According to those observations, A1 carriers would be assumed to show a less pronounced decrease of dopaminergic signaling after negative prediction errors in the indirect pathway and a shift to a more action-oriented behavioral pattern mediated by the direct pathway (Figure 3). Such a pattern bears some resemblance to the concept of behavioral impulsivity (Tomie et al., 1998; Flagel et al., 2010, 2011), and it is noteworthy in this context that the A1 allele has been linked to risk for impulsivity-related psychiatric disorders, most prominently alcohol dependence (Noble et al., 1991; Comings et al., 1996; Noble, 2003; Eisenberg et al., 2007; Wang et al., 2013). However, this does not explain, why A1 carriers exhibit a relatively specific performance disadvantage in the *no-go to win*, but not in the *no-go to avoid losing* condition. One possible reason would be that a punishment instead of a neutral feedback in the *no-go to avoid losing* condition might lead to a higher prediction error as compared to a neutral feedback instead of a reward in the *no-go to win* condition. Another reason might be that, for example, serotonin plays a specific role in punishment-related behavior (Daw et al., 2002; Boureau and Dayan, 2011; Cools et al., 2011; Guitart-Masip et al., 2012b, 2013; Den Ouden et al., 2013) and thus further modulates the performance in the *no-go to avoid losing* condition.

The investigation of modulators of stereotyped hard-wired behavioral responses is of interest to clinicians as it may help to develop novel treatment approaches for neurological or psychiatric disorders. The TaqIA polymorphism is one of the most extensively studied genetic variations in neuropsychiatric disorders with presumed dopaminergic dysfunction, and studies have pointed to a potential pleiotropic effect with A1 allele carriers showing an increased risk for addiction, but a lower risk for schizophrenia (e.g., Comings et al., 1996; Noble, 2003; Dubertret et al., 2004; Wang et al., 2013; Zhang et al., 2014). Moreover, studies in healthy humans have suggested a role of the TaqIA A1 variant in approach-related personality traits (Noble et al., 1998; Reuter et al., 2006; Lee et al., 2007; Smillie et al., 2010) and on motivated interference processing (Richter et al., 2013). The relation between the single nucleotide polymorphism (SNP) and instrumental learning has also been investigated. Previous studies have shown an impairment of the carriers of the A1 allele in no-go learning to avoid behaviors that yield negative outcomes (Klein et al., 2007; Frank and Hutchison, 2009; Jocham et al., 2009). However, those studies have only used conditions in which participants had to approach a reward or avoid a punishment. Since the interaction between action and valence has a pivotal influence on instrumental learning (Guitart-Masip et al., 2012b), such studies could not provide information on possible action by valence interactions, and the use of the valenced go/no-go-learning task with orthogonalized action and valence enables a more precise investigation of the contribution of the dopaminergic system in behavioral adaptation.

The TaqIA polymorphism, initially identified to be located on the DRD2 gene on human chromosome 11q22–23 (Grandy et al., 1989), is located 10kb downstream of the DRD2 termination codon on 11q23.1, within coding region of the adjacent ankyrin repeat and kinase domain containing 1 (ANKK1) gene (Dubertret et al., 2004; Neville et al., 2004). Because the DRD2 and ANKK1 genes are closely linked (Neville et al., 2004; Ponce et al., 2009), it has been proposed that genetic variations in linkage disequilibrium (LD) with the SNP might explain the observed relationship between the TaqIA and alterations of human dopaminergic neurotransmission. The SNP is indeed in LD with several polymorphisms on the DRD2 gene (Duan et al., 2003; Ritchie and Noble, 2003; Fossella et al., 2006) and one of them is the C957T polymorphism (rs6277) for which also modulations on instrumental learning have been observed (Frank et al., 2007, 2009; Frank and Hutchison, 2009). However, its influence on dopaminergic neurotransmission is not clear since *in vivo* and *in vitro* data are in conflict (Duan et al., 2003; Hirvonen et al., 2004; see also erratum by Hirvonen et al., 2004, 2009a,b) and no association was found between C957T and DA synthesis capacity *in vivo* (Laakso et al., 2005) and C957T and D2 receptor mRNA expression in *post mortem* brain tissue (Zhang et al., 2007). When controlling for a potential influence of this SNP in our analysis, the effect of TaqIA genotype was still significant. We cannot rule out, though, that another variant in the DRD2 gene—or perhaps in the ANKK1 gene—linked to TaqIA might be responsible for the observed genotype-related differences in learning rate.

In order to control for genetic influences of another genetic variant known to affect prefrontal DA levels and thereby cortical D1 receptor stimulation (Gogos et al., 1998; Matsumoto et al., 2003; Meyer-Lindenberg et al., 2005; Slifstein et al., 2008) we selected our participants to have comparable distributions of the COMT Val108/158 Met genotype. Importantly, the allelic distribution of COMT Val108/158 Met alleles did not differ significantly between TaqIA A1 carriers and A2 homozygotes.

It must nevertheless be kept in mind that genetic variations within the dopaminergic system do not exert their effects in isolation. Frank et al. (2007), for example, observed multiple roles for DA in reinforcement learning when investigating effects of the COMT Val108/158 Met, the DARPP-32, and the DRD2 C957T polymorphism on reward-based probabilistic learning. Even though we controlled for these polymorphisms in our experiment, we cannot completely rule out gene-gene interactions. Our moderately large sample sizes allowed us to examine effects of single genetic variants on behavioral outcomes, but the systematic analysis of gene-gene interactions would require substantially larger cohorts. In addition to the likely polygenic contribution of variants in the dopaminergic system to action by valence interaction, also other neuromodulatory transmitters must be considered in future studies.

## Conclusion

Our findings provide further evidence for a potential genetic basis of individual differences in probabilistic learning and, more specifically, suggest that genetically mediated differences in dopaminergic neuromodulation not only affect learning *per se*, but also can specifically affect behavioral phenomena like a Pavlovian action bias when a reward is expected. With respect to future research directed at individual differences in learning, our findings should thereby caution researchers to take into account the non-orthogonal nature of action by valence interactions.

### Conflict of interest statement

The authors declare that the research was conducted in the absence of any commercial or financial relationships that could be construed as a potential conflict of interest.
