# Corrigendum: Valenced action/inhibition learning in humans is modulated by a genetic variant linked to dopamine D2 receptor expression

**DOI:** 10.3389/fnsys.2015.00036

**Published:** 2015-03-11

**Authors:** Anni Richter, Marc Guitart-Masip, Adriana Barman, Catherine Libeau, Gusalija Behnisch, Sophia Czerney, Denny Schanze, Anne Assmann, Marieke Klein, Emrah Düzel, Martin Zenker, Constanze Seidenbecher, Björn H. Schott

**Affiliations:** ^1^Department of Neurochemistry and Molecular Biology and Department of Behavioral Neurology, Leibniz Institute for NeurobiologyMagdeburg, Germany; ^2^Wellcome Trust Centre for Neuroimaging, Institute of Neurology, University College LondonLondon, UK; ^3^Ageing Research Centre, Karolinska InstituteStockholm, Sweden; ^4^Institute of Human Genetics, Otto von Guericke UniversityMagdeburg, Germany; ^5^Institute of Cognitive Neurology and Dementia Research, Otto von Guericke UniversityMagdeburg, Germany; ^6^Institute of Cognitive Neuroscience, University College LondonLondon, UK; ^7^German Center for Neurodegenerative DiseasesMagdeburg, Germany; ^8^Center for Behavioral Brain Sciences, Otto von Guericke University of MagdeburgMagdeburg, Germany; ^9^Department of Psychiatry, Charité University HospitalBerlin, Germany; ^10^Department of Neurology, Otto von Guericke UniversityMagdeburg, Germany

**Keywords:** dopamine D2 receptor, TaqIA, reward learning, motivated learning, action bias

We observed some errors that occurred during the genotyping of DARPP-32 rs907094. Naming of CC and TT homozygotes was swapped, and, furthermore, six genotypes were wrongly identified (three people changed from CT to CC, two people changed from CT to TT, and one person changed from TT to CT). All statistics that included DARPP-32 rs907094 genotype were recomputed. We have corrected the text in the corresponding text passages of the manuscript accordingly (last paragraph of the Results Section and Table 2). Importantly, these corrections did not affect our main findings, the effects attributable to the DRD2 TaqIA polymorphism.

Find below the last paragraph of the Results Section and Table 2 with the corrected statistics including DARPP-32 rs907094 genotype.

**Corrected version of the last paragraph of the Results Section**

Because the TaqIA polymorphism is located downstream of the DRD2 gene, the observed genotype effects might putatively result from linkage disequilibrium with other DRD2 polymorphisms, including the C957T. We indeed observed an imbalanced distribution of the C957T polymorphism (rs6277) among TaqIA A1 carriers vs. A2 homozygotes numerically in the first cohort (χ^2^ = 4.04, *p* = 0.132) and significantly in the second cohort (χ^2^ = 25.49, *p* < 0.001). Moreover, the DARPP-32 polymorphism (rs907094) was unequally distributed in the second cohort only (χ^2^ = 7.62, *p* = 0.022). In order to rule out confounding effects, we included the polymorphisms as covariates in an additional ANCOVA. The same was done for COMT Val108/158Met (rs4680), because the cohorts were stratified with respect to that polymorphism. Importantly, the fourfold action by valence by time by genotype interaction for the TaqIA polymorphism remained significant [cohort 1: *F*_(1, 82)_ = 4.67, *p* = 0.034, cohort 2: *F*_(1, 90)_ = 4.65, *p* = 0.034], while there was no effect for C957T (cohort 1: *p* = 0.484, cohort 2: *p* = 0.832), DARPP-32 (cohort 1: *p* = 0.610, cohort 2: *p* = 0.235), or COMT Val108/158Met polymorphism (cohort 1: *p* = 0.149, cohort 2: *p* = 0.842).

**Corrected version of Table 2**.

**Table 2:** **Demographic data**.

	**A1+**	**A1−**	
**COHORT 1**			
Women/Men (*n* = 87)	17/20	26/24	χ^2^ = 0.31, *p =* 0.577
Mean age (*n* = 87)	24.9 ± 3.6	24.3 ± 2.6	*t*_(85)_ = 0.83, *p* = 0.410
Smokers/Nonsmokers (*n* = 87)	15/22	14/36	χ^2^ = 1.51, *p* = 0.220
COMT mm/vm/vv (*n* = 87)	13/14/10	18/15/17	χ^2^ = 0.73, *p* = 0.694
DAT1-VNTR 9+/9− (*n* = 85)	11/25	15/34	χ^2^ < 0.01, *p* = 0.996
C957T CC/CT/TT (*n* = 87)	11/19/7	8/24/18	χ^2^ = 4.04, *p* = 0.132
DARPP-32 CC/CT/TT (*n* = 87)	4/13/20	3/18/29	χ^2^ = 0.68, *p* = 0.714
**COHORT 2**			
Women/Men (*n* = 95)	13/21	35/26	χ^2^ = 3.20, *p* = 0.074
Mean age (*n* = 95)	25.2 ± 3.3	24.2 ± 2.4	*t*_(93)_ = 1.58, *p* = 0.121
Smokers/Nonsmokers (*n* = 95)	5/29	14/47	χ^2^ = 0.93, *p* = 0.335
COMT mm/vm/vv (*n* = 95)	11/14/9	19/27/15	χ^2^ = 0.09, *p* = 0.957
DAT1-VNTR 9+/9− (*n* = 93)	17/17	32/27	χ^2^ = 0.16, *p* = 0.693
C957T CC/CT/TT (*n* = 95)	15/17/2	3/37/21	χ^2^ = 25.49, *p* < 0.001
DARPP-32 CC/CT/TT (*n* = 95)	3/15/16	0/20/41	*χ ^2^* = 7.62, *p* = 0.022

*Gender distribution, age (means ± standard deviations), number of smokers and nonsmokers. Allelic distributions for following polymorphisms: COMT Val108/158Met (mm, met homozygotes; vm, val/met heterozygotes; mm, met homozygotes), DAT1-VNTR (9+: carriers of the 9-repeat allele 9/9 and 9/10; 9−: 10-repeat homozygous subjects 10/10), C957T (CC/CT/TT carriers), and DARPP-32 (CC/CT/TT carriers). A1+, carriers of the A1 allele; A1−, A2 homozygotes*.

## Conflict of interest statement

The authors declare that the research was conducted in the absence of any commercial or financial relationships that could be construed as a potential conflict of interest.

